# Optimization of deacidification for concentrated grape juice

**DOI:** 10.1002/fsn3.1037

**Published:** 2019-05-01

**Authors:** Ning Li, Yue Wei, Xuemeng Li, Jiahui Wang, Jiaqian Zhou, Jie Wang

**Affiliations:** ^1^ College of Food Science and Technology Agricultural University of Hebei Baoding China; ^2^ Hebei Agricultural Products Processing Engineering Technology Research Center Baoding China; ^3^ College of Science and Technology Agricultural University of Hebei Huanghua China

**Keywords:** anion‐exchange resin, concentrated grape juice, optimized deacidification, tartaric acid

## Abstract

Excessive organic acids in grape juice will not only result in poor taste but will also cause turbidity and sedimentation. Tartaric acid exerts the most significant acidity among all organic acids in grape juice. In this study, we used tartaric acid as the main target and anion‐exchange resin to remove tartaric acid from concentrated grape juice. Factors influencing the removal process were optimized by liquid chromatography with ultraviolet detection and statistical analysis for optimal deacidification of concentrated grape juice. Use of the anion‐exchange resin 335 treat the concentrated grape juice at a ratio of 1:6 (2:11.98) at 15.57°C for 4.35 hr. The tartaric acid removal rate reached 69.01%; the anion‐exchange resin 335 demonstrated the best removal effect.

## 1 INTRODUCTION

Grapes are one of the largest and common fruit crops with high antioxidant capacity worldwide. Grapes contain a large amount of active ingredients, including flavonoids (Ali, Badr El‐Din, & Abou‐El‐magd, 2015), polyphenols (Antoniolli, Fontana, Piccoli, & Bottini, 2015), anthocyanins (Fernández‐Marín, Guerrero, Puertas, García‐Parrilla, & Cantos‐Villar, 2013), and resveratrol (Shrikanta, Kumar, & Govindaswamy, 2015), which are found primarily in the skin and seeds. The beneficial effects of grape seeds and skin on human or animal health can be attributed to their antioxidant, anti‐inflammatory, anticancer, and antibacterial activities (Perumalla & Hettiarachchy, 2011; Toaldo et al., 2013). Therefore, grapes have high nutritional and medicinal value.

Concentrated grape juice, a processed drink made from grapes, is convenient to drink and has high nutritional and economic value. Tartaric, malic, and citric acids are important organic acids in grape cultivars (Öncül & Karabıyıklı, 2016). Among them, tartaric and malic acids are the most important (Pavloušek & Kumšta, 2011) and play an important role in manufacturing concentrated grape juice because they affect the color, flavor, and stability of the juice (Preiner et al., 2013). The degree of acidity of concentrated grape juice is higher than the generally accepted degree of juice acidity; as such, a deacidification step is used to reduce the acidity in the production process (Preiner et al., 2013). Malic acid has relatively active physiological metabolism and can be easily removed during subsequent processing (Volschenk & Van Vuuren, 2006). Tartaric acid has strong acidity but cannot be easily removed. This acid easily forms calcium tartrate or potassium hydrogen tartrate with metal ions (Guise et al., 2014; Jiang, Nan, & Hua, 2008); the complex causes beverage turbidity, which affects the quality of the processed grape juice. Therefore, reducing the tartaric acid content without affecting the quality of concentrated grape juice is important.

At present, acid reduction methods used for wine or juice mainly include chemical (Vera et al., 2003), physical (Mondor, Ippersiel, & Lamarche, 2012), and biological acid‐reducing methods mainly for malic acid (Viljakainen & Laakso, 2000). Chemical acid‐reducing method mainly utilizes partial alkali salt to react with organic acid in the juice and reduce the acid content. Although this method is rapid and effective and regardless of the chemical reagents used, it poses certain limitations and security risks, which are inconsistent with the consumer's pursuit of natural additive‐free consumer psychology (Vera et al., 2003). Physical acid‐reducing methods mainly include cryogenic freezing, electrodialysis (Kaláb & Palatý, 2012), organic extraction (Marchitan et al., 2010), and ion‐exchange method (Kaya, Şahbaz, Arar, Yüksel, & Yüksel, 2015). Anion‐exchange method is the best wine acid‐reducing method (Kaya et al., 2015). The basic principle of anion‐exchange resin for removing acid from wine or juice involves the ion‐exchange reaction of OH– with the organic acid radical in the wine or juice and the exchange of the organic acid radical onto the resin; the –OH– detached resin enters the solution and undergoes acid–base neutralization reaction with H+ in wine or juice, thereby reducing the acidity of the wine or juice (Zhang, Liu, Peng, & Chen, 2015). Compared with other methods, ion‐exchange method requires low acid‐reducing equipment, has low cost, and does not contaminate the wine and juice (Vibhakar, Prabhakar, & Bhatnagar, 1966). Therefore, in the present study, we used anion‐exchange resin method to deacidify concentrated grape juice.

An effective and accurate detection method must be established to determine tartaric acid removal efficiency. The reported methodologies include capillary electrophoresis (Mato, Suarez‐Luque, & Huidobro, 2007), which presents advantages including high resolution, simplicity, and short analysis time; however, this method also has disadvantages including low reproducibility; gas chromatography, mainly with flame ionization and mass spectrometry (MS) detectors (Fernández‐Fernández et al., 2010); liquid chromatography (LC) with ultraviolet (UV) method (Scherer et al., 2012); and chemiluminescent and electrochemical methods with refractive index, conductivity, and MS detectors (Flores, Hellín, & Fenoll, 2012). LC‐UV method is used for quantitative and qualitative analyses of complex food samples. This method is characterized by its reliability, versatility, high sensitivity, ease of use, simple maintenance, and low equipment production costs (Petrovic & Barceló, 2013). Therefore, LC‐UV method has a wide range of applications in experimental research. In the present experiment, tartaric acid was quantified by LC‐UV method.

This study used tartaric acid as the main target and anion‐exchange resin to deacidify the concentrated grape juice with high acidity. Factors affecting deacidification were optimized to easily deacidify the concentrated grape juice. These factors include amount of resin, treatment temperature, and processing time. This study also examined the influence of deacidification on concentrated grape juice by sensory analysis of the final concentrated grape juice. The deacidification method provides theoretical guidance for reducing the acidity of concentrated grape juice in the industry.

## 2 MATERIALS AND METHODS

### 2.1 Materials

Concentrated grape juice: Natural grape juice obtained from squeezing of Syrah or Shiraz, Cabernet Sauvignon and Malbec grapes was placed in the evaporation equipment, which is a flat, spiral, or tubular heat exchanger (concentrated grape juice is purchased from Shangri‐La Wine Co., Ltd.). The grape juice was heated in the evaporator and concentrated to 40 Brix. The concentrated grape juice was placed in containers and stored in a freezer at 15°C before use (Juchuang Environmental Protection Equipment Co., Ltd., Haier Group Co., Ltd.). After LC‐UV method and calibration of the standard curve were performed, the tartaric acid content in the concentrated grape juice was found to be 17.68%.

Reagents: NaOH, HCl, NaCl, KH_2_PO_4_, and acetonitrile were purchased from Xilong Chemical Co., Ltd. All of the chemicals used were of chromatographic reagent grade. Double‐distilled water was used throughout the study.

The following instruments were used: Waters 1525 Chromatograph and Waters 2489 UV‐detector (Waters Co., Ltd.), 335 anion‐exchange resins, D‐314 anion‐exchange resin, and D‐914 anion‐exchange resin with 150 mm × 4.6 mm and 5 µm particle size (Waters Atlantis T3).

### 2.2 Methods

#### 2.2.1 Grape juice treatment

Because the viscosity of concentrated grape juice is too high to be filtered directly by resin, it must be diluted for a certain number of times before using resin to deacidify. By optimizing the dilution multiple, this study determined that 100 times dilution is the best dilution multiple. The grape juice involved in the 2.2.4 Experimental design section is 100 times diluted grape juice.

A certain amount of activated anion adsorption resin was added to the grape juice and continuously stirred. The resin was allowed to fully adsorb at a certain temperature and then filtered to obtain the supernatant (Kontogiannopoulos, Patsios, & Karabelas, 2016; Yalcin, Ozcalik, Altiok, & Bayraktar, 2008). The concentration of tartaric acid in the filtrate was determined by LC‐UV method with the standard curve of tartaric acid. Tartaric acid removal rate was calculated as follows:

$$\text{Removal rate}=\frac{\text{Content of tartaric acid before deacidification}-\text{Content of tartaric acid after deacidification}}{\text{Content of tartaric acid before deacidification}}\times 100\%$$

#### 2.2.2 Resin pretreatment

The resin was soaked in a 90% ethanol solution for more than 24 hr and mixed to remove aromatic hydrocarbons and alcohol‐soluble substances. The alcohol solution was poured and repeatedly washed with distilled water until the resin removed the ethanol completely. The resin was then filtered and dried.

Alkaline treatment (acid water–alkaline water) was performed, and the resin treated with ethanol was soaked in 5% HCl solution for several hours. The solution was mixed properly. The HCl solution was removed and washed with water to obtain a pH of 4–5. The resin was then soaked in 5% NaOH solution for several hours and mixed properly. The NaOH solution was removed and washed with water until pH 8–9. The resin was soaked in distilled water for storage (Alharati, Swesi, Fiaty, & Charcosset, 2017; Ortega et al., 2017).

#### 2.2.3 LC‐UV condition

Chromatographic column: 150 mm × 4.6 mm and 5 µm particle size (Atlantis T3, Waters)
Mobile phase: 0.02 mol/L KH_2_PO_4_:acetonitrile (V:V = 90:10), pH = 3
Sample: 2 µl
Flow rate: 1.00 ml/min
Column temperature: 30°C
UV wavelength: 210 nm

#### 2.2.4 Experimental design

Many factors affect deacidification; as such, selecting significant factors and optimizing them can increase the degree of deacidification. Kaya et al. (2015) removed tartaric acid by gel and macroporous ion‐exchange resins and studied factors affecting the removal rate; the resin amount and pH considerably influenced the removal rate. Other researchers speculated that initial acid concentration, contact time, and temperature during acid adsorption affected the removal rate of tartaric acid (Uslu, Inci, Bayazit, & Demir, 2009). In the present work, we studied the effect of resin type, amount, adsorption temperature, and adsorption time on tartaric acid removal rate. All experiments were carried out in triplicate.

In brief, 10 g of activated 335 anion‐exchange resin, D‐314 anion‐exchange resin, and D‐914 anion‐exchange resin were used. Each resin was added to 200 ml grape juice. The mixture was stirred thoroughly to ensure sample homogeneity. The solution was allowed to fully adsorb at 20°C for 3 hr and filtered to obtain the supernatant. The removal rate of tartaric acid was calculated to obtain the best adsorption resin for subsequent optimization experiments.

In 200 ml grape juice, add about 10 g of the best‐activated resin, and then stir thoroughly. The best‐activated resin was allowed to fully adsorb at different temperatures (0, 15, 30, 45, and 50°C) for 3 hr and filtered to obtain the supernatant. Higher temperatures were not considered because further heating greatly increases the energy consumption and the risk of quality deterioration of bioactive compounds (Kontogiannopoulos et al., 2016). Finally, the tartaric acid removal rate was calculated, and the temperature corresponding to the maximum removal rate was the optimum adsorption temperature of the resin.

After the optimum adsorption temperature of the resin was determined, the effect of resin quantity on the ability of resin to adsorb tartaric acid was evaluated. Various concentrations of the best‐activated resin (2, 5, 10, and 16 g) were added to 2 ml of the concentrated grape juice and 200 ml of deionized water and then mixed. The solution was allowed to adsorb at 20°C for 3 hr and filtered to obtain the supernatant. The concentration of tartaric acid in the filtrate was determined by the same method as above. The tartaric acid removal rate was calculated to obtain the best amount of additives of the resin.

Resin adsorption time also affects the degree of adsorption of tartaric acid by the resin. In 200 ml grape juice, add about 10 g of the best‐activated resin, and then stir thoroughly. The solution was allowed to fully adsorb at 20°C for 2–6 hr and filtered to obtain the supernatant. Finally, the concentration of tartaric acid in the filtrate was determined. The tartaric acid removal rate was calculated, and the best adsorption time of resin was established.

#### 2.2.5 Statistical analysis

The experimental design for optimizing deacidification for concentrated grape juice was carried out using Design‐expert 8.0.6 while maximizing the removal rate of tartaric acid as the optimization criterion.

According to single‐factor experiments, the first phase used SPSS software to determine factors that primarily influenced the removal rate of tartaric acid. Each variable was evaluated at three levels: high (+1), low (−1), and middle level (0).

In the second phase, according to the first phase results, variables with the most significant effect on the removal rate of tartaric acid were selected using Box–Behnken design and response surface methodology (RSM) to investigate their interaction effects. The Box–Behnken design determined the optimization levels through three major aspects, that is, by performing statistically designed experiments, by estimating coefficients in a mathematical model, and by predicting responses (Gajdhane, Bhagwat, & Dandge, 2016; Ghorbannezhad, Bay, Yolmeh, Yadollahi, & Moghadam, 2016; Zong et al., 2010).

#### 2.2.6 Sensory analysis

Deacidification of concentrated grape juice, which was performed by the optimized deacidification method obtained through the RSM experimental design, was marked as sample A. An appropriate amount of concentrated grape juice, which was not subjected to deacidification under the same conditions, was prepared and used for sensory analysis, and was marked as sample B. The sensory analysis of the concentrated grape juice was conducted in Hebei Agricultural University. Using quantitative descriptive analysis (QDA), ten students with knowledge of sensory analysis were selected for sensory evaluation of samples A and B. The concentrated grape juice was served as 30 ml aliquots in XL5 tasting glasses covered with Petri dishes. Samples should not be swallowed.

The evaluation process consisted of the following steps: Firstly, the representative descriptive vocabulary of samples was screened out by each reviewer. Then, the intensity of each descriptive vocabulary was scaled 0–10, 0 = nonexistence, 5 = medium, and 10 = strong. Finally, the descriptive vocabulary and its grades were used to make QDA. Gathering descriptive vocabulary selected by 10 reviewers made the QDA analysis point table, which is shown in Table 1.

**Table 1:** QDA analysis point table

Descriptive vocabulary	Grades of sample A (0–10)	Grades of sample B (0–10)
Grape flavor	0 □□□□□□□□□□ 10	0 □□□□□□□□□□ 10
Clarity	0 □□□□□□□□□□ 10	0 □□□□□□□□□□ 10
Sweetness	0 □□□□□□□□□□ 10	0 □□□□□□□□□□ 10
Acidity	0 □□□□□□□□□□ 10	0 □□□□□□□□□□ 10
Astringency	0 □□□□□□□□□□ 10	0 □□□□□□□□□□ 10
Color and luster	0 □□□□□□□□□□ 10	0 □□□□□□□□□□ 10
Scent	0 □□□□□□□□□□ 10	0 □□□□□□□□□□ 10

## 3 RESULTS AND DISCUSSION

### 3.1 Adsorption resin selection

**Figure 1:**
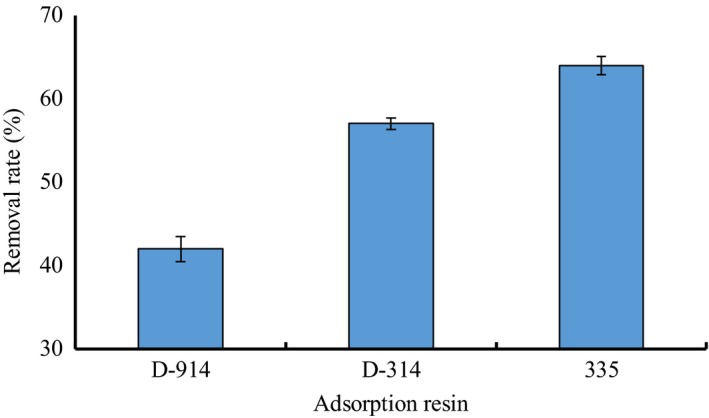
Effects of different resins on the removal rate of tartaric acid

Different types of ion‐exchange resins possess different adsorption capacities. The ability of three resins to adsorb tartaric acid is shown in Figure 1. In addition to D‐914, the two other anion‐exchange resins (335 and D‐314) exhibited good adsorption and were suitable for the deacidification of the concentrated grape juice. The tartaric acid average removal rates of the 335 and D‐314 resins were 64.73% and 57.53%, respectively. Therefore, we selected the 335 resin as the anion‐exchange resin for optimization of the deacidification process.

### 3.2 Resin amount

**Figure 2:**
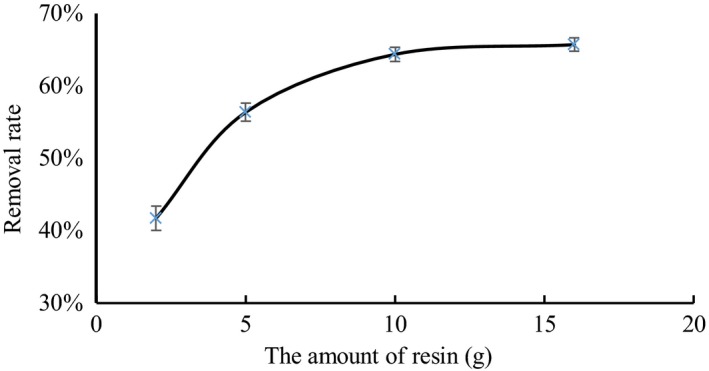
Effects of different amounts of resin on the removal rate of tartaric acid

Figure 2 shows the effect of the resin amount on tartaric acid removal. When the resin amount was increased from 2 to 10 g, the removal rate of tartaric acid increased rapidly. However, when the resin amount exceeded 10 g, the removal rate of tartaric acid increased gradually. When using 10 g of the resin, the tartaric acid removal rate was 64.54% because increasing the amount of resin increased the chance that the resin and tartaric acid come into contact with each other (Namasivayam & Ranganathan, 1995; Shaw, Shah, & Tailor, 2011). Therefore, the greater the amount of resin added, the stronger the ability of the resin to remove tartaric acid will be. Although increasing the amount of resin can increase the adsorption of tartaric acid, the amount of tartaric acid adsorbed on the unit resin decreased. The removal rate of tartaric acid remained basically unchanged when the resin reached the optimum amount. To absorb as much tartaric acid as possible and consider the cost savings, we used 10 g of the resin to absorb tartaric acid by using 1:5 ratio of the concentrated grape juice to resin.

### 3.3 Treatment temperature

**Figure 3:**
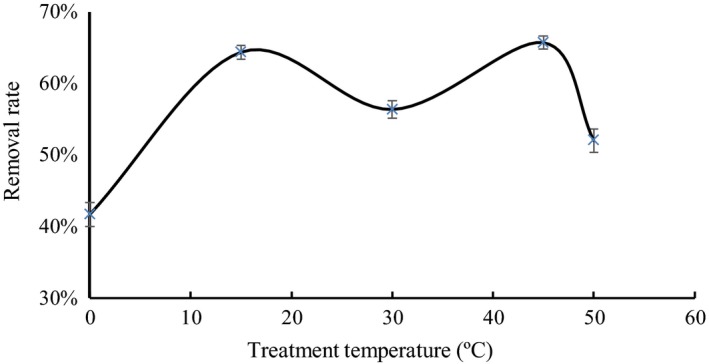
Effects of different treatment temperatures on the removal rate of tartaric acid

The effect of treatment temperature on the removal rate of tartaric acid is plotted in Figure 3. Increasing the temperature can accelerate the thermal motion of molecules, thereby contributing to adsorption. The tartaric acid removal rate reached the maximum at 15°C because tartaric acid precipitation tended to occur at 15°C when nuclei are present in the solution. The presence of the experimental resin served as the core of the nuclei. In addition to the adsorption removal by the resin at 15°C, tartaric acid precipitation contributed to the removal rate (Fan, Ding, & Jiang, 2002).

As the temperature continued to rise, the precipitation of tartaric acid decreased. At 30°C, tartaric acid removal mainly depended on the adsorption of the resin. With further increase in the temperature, the tartaric acid removal rate also increased. At 45°C, the maximum value was reached. At over 45°C, the tartaric acid removal rate decreased. Given that the tartaric acid removal rates at 15 and 45°C were similar and to save heat energy, we used 15°C for tartaric acid precipitation to obtain a high tartaric acid removal rate.

### 3.4 Processing time

**Figure 4:**
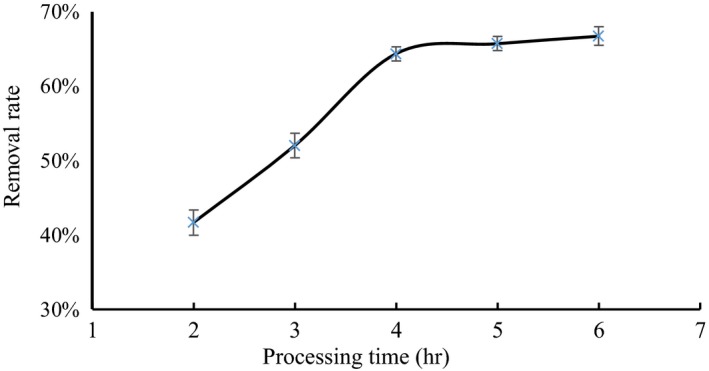
Effects of different processing times on the removal rate of tartaric acid

As shown in Figure 4, the removal rate of tartaric acid increased with processing time. When full contact was reached at 4 hr, the removal rate of tartaric acid remained essentially unchanged; that is, the resin on the adsorption of tartaric acid reached the equilibrium. The process where resin adsorbed tartaric acid was mass transfer. With increasing time, tartaric acid continuously transferred from the solution onto the resin, indicating that the removal rate of tartaric acid increased. When the amount of tartaric acid that transferred from the solution to the resin was equal to the amount of tartaric acid that transferred from the resin to the solution, the adsorption of tartaric acid by the resin reached the equilibrium (Kang, Fang, Wei, & Yang, 2011). As shown in Figure 4, 4 hr was the equilibrium point and determined as the optimum processing time.

### 3.5 Statistical analysis

According to the results of single‐factor experiments, a statistical method was used to determine factors that primarily influenced the removal rate of tartaric acid. According to the center combination experimental design principle of Box–Behnken, three‐factor and three‐level response surface analysis experiments were conducted. Table 2 presents the low (−1), high (+1), and middle concentration (0) levels of each variable. Table 3 shows 17 runs of the Box–Behnken design and RSM and the removal rate of tartaric acid. The experimental results were fitted into a quadratic polynomial equation as follows:

$$Y=+66.75+1.38\text{A}+2.50\text{B}+2.55\text{C}+1.40\text{AB}+0.40\text{AC}+\;0.17\text{BC}-1.45{\text{A}}^{2}-2.43{\text{B}}^{2}-1.41{\text{C}}^{2}$$

where *Y* represents the removal rate of tartaric acid; and A, B, and C correspond to resin amount, treatment temperature, and processing time, respectively.

**Table 2:** Level of variables used for trials

Variables	Low (−1)	Middle (0)	High (+1)
Resin (g)	8	10	12
Temperature (°C)	13	15	17
Time (hr)	3.5	4.0	4.5

**Table 3:** Box–Behnken design showed coded values of independent variables with observed removal rate of tartaric acid; each row corresponds to a single experiment

Runs	Resin (g)	Temperature (°C)	Time (hr)	Removal rate of tartaric acid (%)		
				Actual	Predicted	Residual
1	−1	−1	0	60.23	60.40	−0.17
2	1	−1	0	60.63	60.35	0.28
3	−1	1	0	62.30	62.59	−0.29
4	1	1	0	68.32	68.14	0.18
5	−1	0	−1	60.94	60.38	0.56
6	1	0	−1	62.42	62.32	0.10
7	−1	0	1	64.56	64.66	−0.10
8	1	0	1	67.66	68.22	−0.56
9	0	−1	−1	57.65	58.04	−0.39
10	0	1	−1	62.41	62.69	−0.28
11	0	−1	1	63.07	62.79	0.28
12	0	1	1	68.51	68.12	0.39
13	0	0	0	67.82	66.75	1.07
14	0	0	0	67.55	66.75	0.80
15	0	0	0	67.02	66.75	0.27
16	0	0	0	66.43	66.75	−0.32
17	0	0	0	64.91	66.75	−1.84

**Figure 5:**
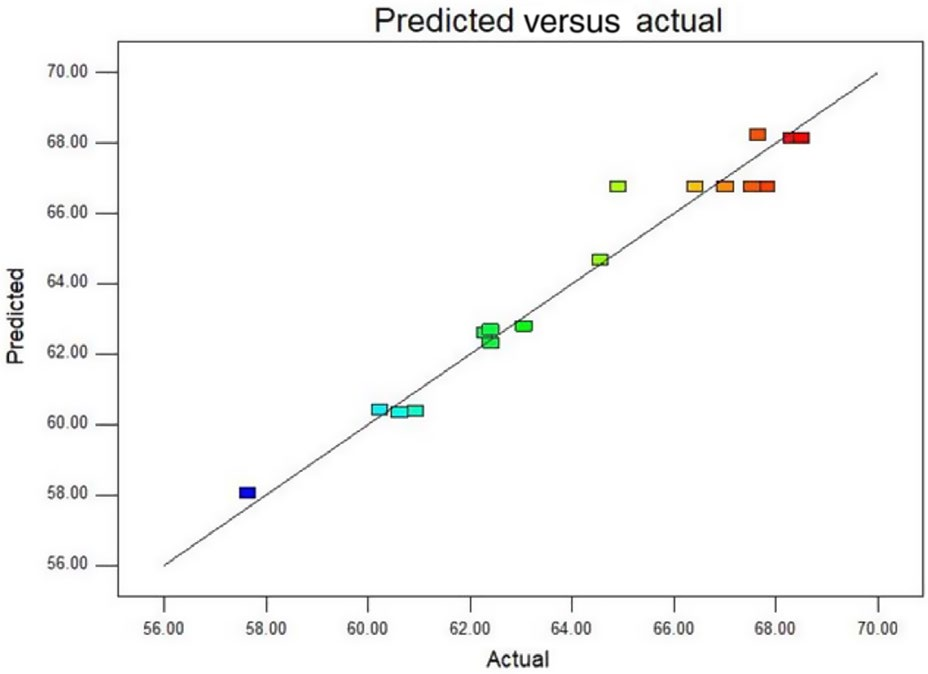
Plot of predicted and actual values for removal rate of tartaric acid

Figure 5 shows the actual values for the removal rate of tartaric acid and the predicted values determined by the model equation. The experimental data for the removal rate of tartaric acid were statistically analyzed by ANOVA (Table 4). The ANOVA of the second‐order quadratic polynomial model for response showed that the model *F*‐value of 20.03 implies that the model was significant. The chance that a “Model *F*‐Value” this large could occur due to noise is only 0.03%. The complex correlation coefficient *R* ^2^ = 96.26% indicates that 96.26% of the change in the response value originates from the selected variables. The *p*‐value of lack of fit was >0.05, indicating that the lack of fit was insignificant. A relatively lower value of the coefficient of variation (CV = 1.52%) indicated the good precision and reliability of the experiment.

**Table 4:** Analysis of variance of regression equation (Box–Behnken design)

Source	Sum of Squares	Degree of freedom	Mean Square	*F*‐value	*p*‐value Prob > *F*
Model	171.96	9	19.11	20.03	0.0003
A‐Resin	15.13	1	15.13	15.86	0.0053
B‐Temperature	49.80	1	49.80	52.20	0.0002
C‐Time	51.92	1	51.92	54.42	0.0002
AB	7.90	1	7.90	8.28	0.0238
AC	0.66	1	0.66	0.69	0.4343
BC	0.12	1	0.12	0.12	0.7380
A^2^	8.80	1	8.80	9.22	0.0189
B^2^	24.87	1	24.87	26.07	0.0014
C^2^	8.32	1	8.32	8.72	0.0213
Residual	6.68	7	0.95		
Lack of fit	1.33	3	0.44	0.33	0.8040
Pure error	5.35	4	1.34		
Cor total	178.64	16			

Note

AB, AC, and BC represent interaction effects of variables A, B, and C; A^2^, B^2^, and C^2^ correspond to squared effects of variables.

**Figure 6:**
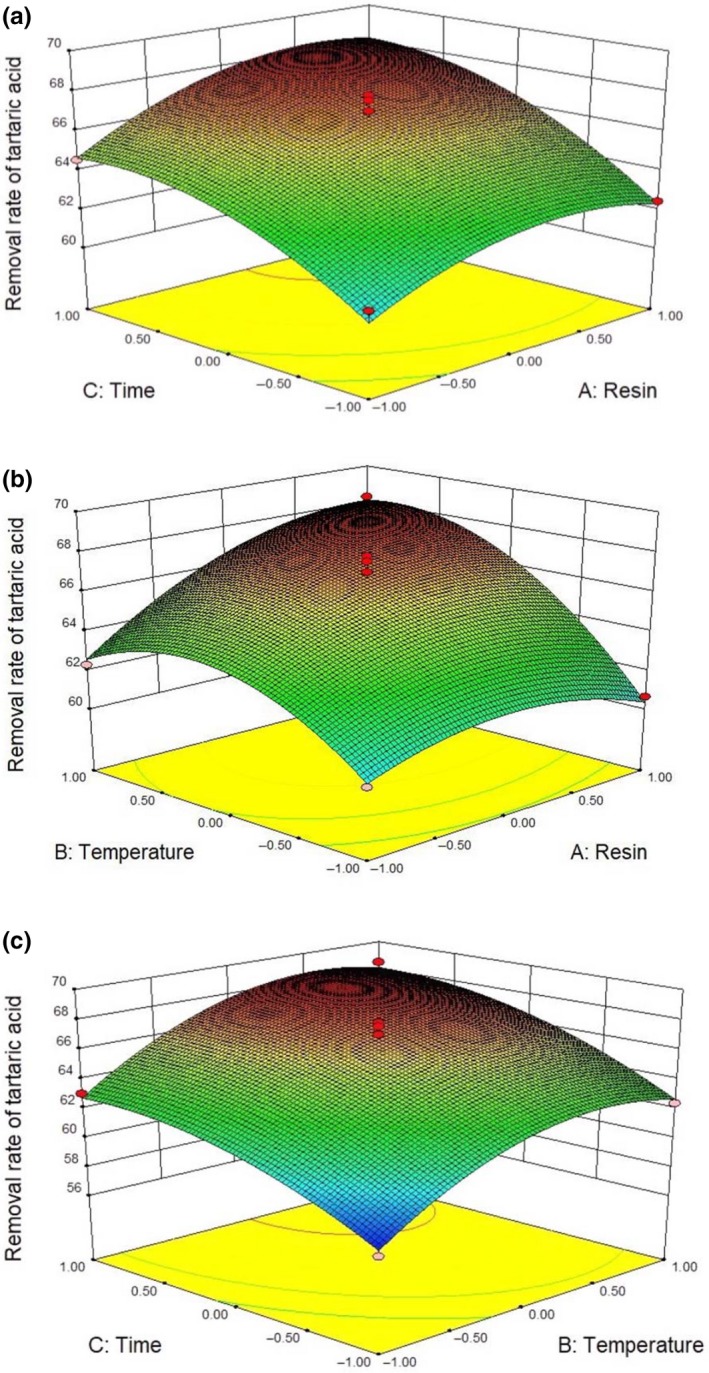
Response surface contour plots of the removal rate of tartaric acid for two independent variables. (a) Three‐dimensional plots for resin amount and treatment temperature while processing time constant at 4 hr. (b) Three‐dimensional plots for resin amount and processing time while treatment temperature constant at 15°C. (c) Three‐dimensional plots for treatment temperature and processing time while resin amount constant at 10 g

The three‐dimensional response plot is shown in Figure 6. Each response surface represented the interaction between the two independent variables when one variable was at the optimal level. Figure 6a plots the interaction effect of resin amount and treatment temperature on the removal rate of tartaric acid. The removal rate of tartaric acid increased with increasing resin amount (2–16 g) and treatment temperature (0–15°C). Afterward, the removal rate slightly increased with resin amount of 10–16 g and decreased at treatment temperature within 15–30°C. Figure 6b represents the interaction between resin amount and processing time. Figure 6c depicts the interaction between treatment temperature and processing time. Figure 6b,c displays similarity with Figure 6a. The optimal conditions of A, B, and C, which were analyzed by Design‐expert 8.0.6, were 11.98 g, 15.57°C, and 4.35 hr, respectively, and the obtained removal rate of tartaric acid was 69.01%. After verification by experiment, the actual value is not considerably different from the predicted result. Hence, the result analyzed by Design‐expert 8.0.6 had high credibility.

### 3.6 Sensory analysis

**Figure 7:**
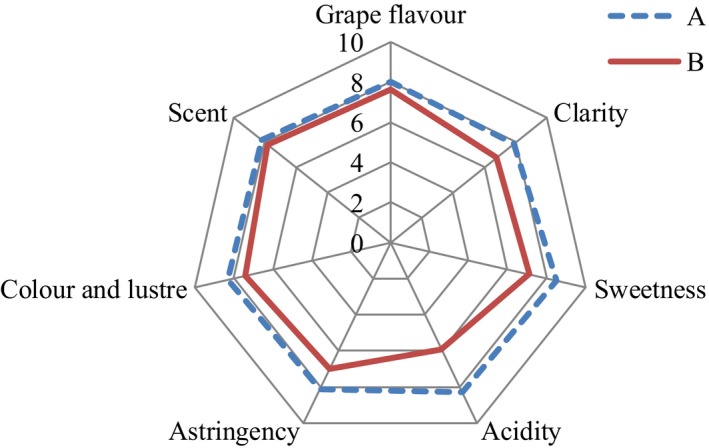
Cobwebs QDA data of concentrated grape juice. A, The deacidified concentrated grape juice; B, The nondeacidified concentrated grape juice

Gathering descriptive vocabulary selected by ten reviewers made the QDA analysis point table, which is shown in Table 1. Table 5 shows grades of the descriptive vocabulary. Figure 7 is a spider QDA diagram based on the data of Table 5. The data in Figure 7 are more visualized. The intensity of each characteristic expressed by the two concentrated grape juices on the same organoleptic properties was different. Through comparison of A and B, we found that the clarity, sweetness, acidity, and astringency as well as color and luster of A were slightly better than B. The other sensory characteristics showed minimal difference. Therefore, the concentrated grape juice treated by the optimized deacidification method exhibited better taste than the concentrated grape juice that was not subjected to deacidification treatment.

**Table 5:** Sensory characteristics of strength evaluation results

Sample	No.	Grape flavor	Clarity	Sweetness	Acidity	Astringency	Color and luster	Scent
A	1	8	7	8	9	8	8	9
	2	7	8	9	7	9	9	8
	3	9	9	8	8	9	8	8
	4	8	8	10	9	7	9	9
	5	7	7	9	9	8	7	7
	6	8	8	7	8	8	8	8
	7	9	9	8	8	9	8	8
	8	9	7	7	8	8	9	8
	9	8	8	9	9	8	8	9
	10	7	8	10	8	7	9	8
	The average grade	8	7.9	8.5	8.3	8.1	8.3	8.2
B	1	7	6	7	6	7	8	8
	2	7	6	6	5	6	7	7
	3	8	7	7	6	8	7	9
	4	9	8	6	5	8	9	8
	5	7	7	8	7	6	6	8
	6	6	6	8	8	7	7	7
	7	9	7	7	5	7	7	8
	8	8	7	9	6	8	7	8
	9	8	8	6	6	6	8	7
	10	7	6	7	5	7	8	8
	The average grade	7.6	6.8	7.1	5.9	7.0	7.4	7.8

Note

A, The deacidified concentrated grape juice; B, The nondeacidified concentrated grape juice.

## 4 CONCLUSION

This study showed that resin type, dosage, processing temperature, and processing time exhibited certain effects on the removal rate of tartaric acid. Among the resins evaluated, the 335 resin demonstrated the best removal effect on tartaric acid and was used to treat the concentrated grape juice at a ratio of 1:6 (2:11.98) at 15.57°C for 4.35 hr. The tartaric acid removal rate reached 69.01%, and the concentrated grape juice obtained by the optimized deacidification method had better flavor. In this study, a method of reducing tartaric acid in concentrated grape juice by anionic resin was established, and the quality of concentrated grape juice was improved. This method can be widely used in industrial production.

## CONFLICT OF INTEREST

The authors declare that they have no conflicts of interests.

## ETHICAL REVIEW

This study does not involve any human or animal testing.

## INFORMED CONSENT

Written informed consent was obtained from all study participants.
